# Patch Materials for Pulmonary Artery Arterioplasty and Right Ventricular Outflow Tract Augmentation: A Review

**DOI:** 10.1007/s00246-023-03152-7

**Published:** 2023-05-07

**Authors:** William E. Schwartzman, Michael Jimenez, Andrew R. Yates, Aimee K. Armstrong, Arash Salavitabar, Kan K. Hor, Simon Hoerstrup, Maximilian Y. Emmert, Toshiharu Shinoka, Sergio A. Carrillo, Christopher K. Breuer, John M. Kelly

**Affiliations:** 1 The Ohio State University College of Medicine, Columbus, OH USA; 2 The Heart Center, Nationwide Children’s Hospital, Columbus, OH USA; 3 Department of Pediatrics, The Ohio State University College of Medicine, Columbus, OH USA; 4 Center for Regenerative Medicine, Abigail Wexner Research Institute at Nationwide Children’s Hospital, Columbus, OH USA; 5 Department of Surgery, The Ohio State University Wexner Medical Center, Columbus, OH USA; 6 Department of Cardiothoracic Surgery, Nationwide Children’s Hospital, Columbus, OH USA; 7 Institute for Regenerative Medicine, University of Zurich, Zurich, Switzerland; 8 Department of Cardiothoracic and Vascular Surgery, German Heart Center Berlin, Berlin, Germany; 9 Department of Cardiovascular Surgery, Charité Universitätsmedizin Berlin, Berlin, Germany

**Keywords:** Right ventricular outflow tract, Pulmonary artery, Arterioplasty, Patch

## Abstract

Patch augmentation of the right ventricular outflow tract (RVOT) and pulmonary artery (PA) arterioplasty are relatively common procedures in the surgical treatment of patients with congenital heart disease. To date, several patch materials have been applied with no agreed upon clinical standard. Each patch type has unique performance characteristics, cost, and availability. There are limited data describing the various advantages and disadvantages of different patch materials. We performed a review of studies describing the clinical performance of various RVOT and PA patch materials and found a limited but growing body of literature. Short-term clinical performance has been reported for a multitude of patch types, but comparisons are limited by inconsistent study design and scarce histologic data. Standard clinical criteria for assessment of patch efficacy and criteria for intervention need to be applied across patch types. The field is progressing with improvements in outcomes due to newer patch technologies focused on reducing antigenicity and promoting neotissue formation which may have the ability to grow, remodel, and repair.

**Supplementary Information:**

The online version contains supplementary material available at 10.1007/s00246-023-03152-7.

## Introduction

Clinically significant congenital heart defects (CHD) and vascular anomalies affect nearly 1% of live births [6]. One-third of these cases exhibit life-threatening complications without surgical interventions early in life [7, 8]. For those requiring surgery, implantation of patch material to augment hypoplastic or replace absent structures is common [9]. The ideal patch would repair or replace both the form and function of the deficient or malformed structure. Currently, no such technology exists. However, progress has been made in the development of more effective biomaterials, and important lessons have been learned from their clinical application. Typical patch materials are generally of biological or synthetic origin. Biological patches include those directly sourced from the patient (autografts), from cadavers (allografts), and from other species (xenografts). Tissue-engineered approaches vary significantly in methodology but generally comprise a combination of biological and synthetic approaches to construct new tissue either in vivo or in situ. The archetype patch material must be strong enough to provide adequate suture retention and burst pressure, have favorable handling characteristics to allow for appropriate tissue reconstruction, and be non-thrombogenic while providing appropriate hemostasis. In the long term, it must elicit an immunologically favorable reaction devoid of a persistent foreign body response and lack of significant calcification, which can result in stenosis and reduced tissue elasticity [9–13]. Ideally, patch materials would allow for endothelization, native tissue ingrowth, neovascularization, and improve long-term remodeling to reduce the arrhythmogenic and infectious risks and allow for growth potential [9, 12–14].

The primary intent of this review is to define the reported clinical outcomes of cardiac patch materials applied to the reconstruction of the right ventricular outflow tract (RVOT) and patch arterioplasty of the pulmonary arteries. Tetralogy of Fallot (TOF) is the predominant congenital cardiac lesion requiring patch augmentation of the RVOT, while peripheral pulmonary artery stenosis occurs in 2–3% of patients with congenital heart disease [15]. It can be isolated or associated with congenital heart lesions, including TOF, truncus arteriosus (TA), and valvular pulmonary stenosis or pulmonary atresia [16]. Complications related to current patch materials include stenosis, aneurysm, thrombosis, and lack of growth capacity, which can necessitate subsequent intervention. Outcomes in this review are organized with respect to patch type. A brief overview of each patch is provided to define the purported benefits and shortcomings of each patch material. The analysis of this cohort is complicated by a mixed group of congenital heart lesions with associated congenital and acquired pulmonary artery stenosis, a variability of applied surgical techniques, and significant variation in the study designs, including time to follow-up, definition of criteria for intervention, and determination of patch-related complications and performance. The goal is to provide a clear depiction of the various patch types that have been applied clinically and their associated outcomes. We hope this knowledge serves to inform readers as to the benefits and limitations of current patch technology and the challenges that lay ahead in the development and evaluation of patch materials.

## Methods

An electronic search was performed by two investigators (WS, MJ) in PubMed from the inception of the database until October 2022. We searched for relevant studies with the use of the search terms “RVOT patch,” “right ventricular outflow tract patch,” and “pulmonary artery patch arterioplasty.” A total of 209 results were returned for “RVOT patch,” 818 results for “right ventricular outflow tract patch,” and 47 results for “pulmonary artery patch arterioplasty.” Of those, 38 studies provided sufficient detail to evaluate clinical performance of the patch material of interest. Supplemental Fig. 1A shows the number of studies by year and Supplemental Fig. 1B shows the first reported results of each patch type. Additionally, the index of relevant articles was searched to evaluate other pertinent publications which may not have been discovered in the initial electronic database search. Valved patches and conduits were excluded from this review.

**Fig. 1:**
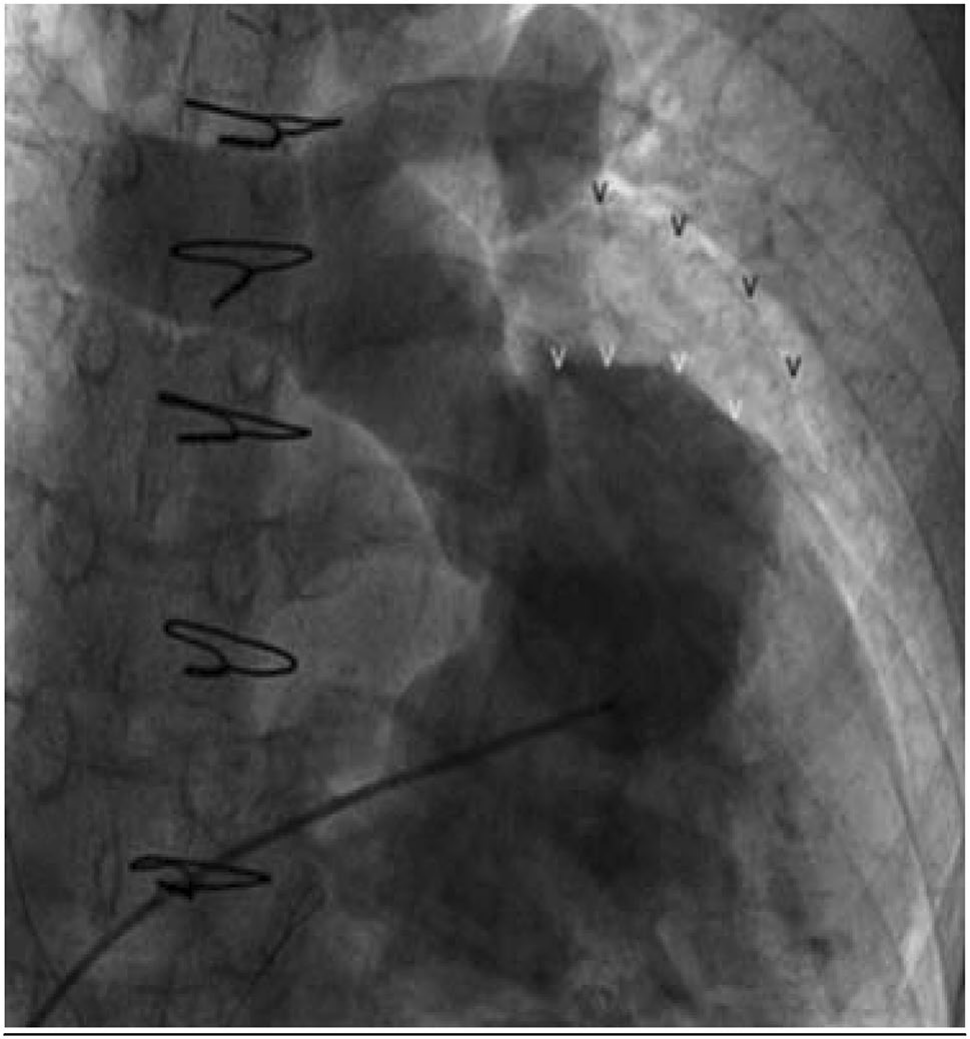
Angiogram demonstrating RVOT aneurysm, a potential complication for patches utilized in RVOT reconstruction [4]

## Results

Detailed analysis of the reported clinical outcomes across patch materials is shown in Table 1. Data are organized with respect to the type of patch material studied, patch-related complications, including need for intervention or replacement, histology, study design, patient population/location of patch implantation, number of patients studied, time to follow-up, and criteria for intervention.

**Table 1:** Summary of clinical studies including type of patch material studied, patch-related complications, mortality, criteria for reintervention, histology, study design, patient population/location of patch implantation, number of patients studied, time to follow-up, and criteria for reintervention

Author	Year	Type of study (pro/ret)	Patch location/operation	Patch material	Cohort	Follow-up time	Criteria for reintervention	Patch-related reintervention/reoperation rates	Additional outcomes	Reported mortality	Histology
Hawe	1970	Retrospective	TOF Repair, RVOT patch	Teflon, Ivalon, AP	202 patients	–	Various	–	Incidence of Aneurysm Formation or Significant Bulging: –Ivalon: 33% –Teflon: 9% –AP: 8%	Hospital mortality 9% (18/202) Grouped by Era: 1957–1962: 20% (16/81) 1963–1967: 2% (2/121) Not distinguished	No
Messina	1994	Prospective	TOF Repair, RVOT patch	GA-treated AP, Saline-Treated AP	36 patients	Avg 5.95 months	–	0%	At 6-month follow-up, patients with saline-treated patches had larger RVOT dimensions than patients with GA-treated patches (p < 0.001)	0%	No
D’Andilli	2005	Prospective	PA reconstruction SVC reconstruction	GA-treated AP	5 patients	12 months	–	0%	–	0%	No
Talwar	2017	Prospective	TOF repair RVOT patch	PTFE GA-fixed AP	103 patients	Mean 6.02-day PTFE 5.89-day GA-fixed AP	Increased mediastinal drainage	10 re-explorations –PTFE: 4 patch-related reinterventions (7 total) –AP: 3 total reinterventions, not distinguished	–	1 patient, excluded from study	No
Ebert	2021	Retrospective	PA reconstruction	Homograft, Bovine Pericardium, AP, CorMatrix	214 patches in 180 patients	Median 3.72 (0.01, 15.69) yrs	–peak gradients > 50 mmHg –symptoms –evidence of severe stenosis (if no available gradient)	34 total (15.9%): 18 surgical and 16 catheter Not distinguished	Found preoperative renal failure (*p* < 0.001) and low weight at surgery (*p* = 0.004) to be associated with reintervention	Discharge Survival: 98.3% Survival to study closure: 94.4% Not distinguished	No
Cresalia	2018	Retrospective	Patch arterioplasty for PA stenosis	Homograft, ePTFE, AP, CorMatrix, Bovine jugular vein	135 patients	Mean: 4.9 yrs (15 days, 11 yrs), median: 4.0 years	PA stenosis, various	10-year reintervention rate: 54% Reintervention for recurrent PA stenosis: 33% Not distinguished	Found weight at operation < 5.1 and age < 30 days both significant predictors of reintervention	30-day survival: 96%, all 6 deaths unrelated to PA plasty 9 deaths in follow-up Not distinguished	No
Rosenthal	1972	Retrospective	TOF repair RVOT patch	AP	135 patients	Mean: 41 (14, 128) months for aneurysm patients –nothing reported for the cohort as a whole	Aneurysm	7 (5%) patients required surgical or catheter aneurysm repair Not distinguished	- 8 (6%) patients developed a patch aneurysm	1 due to false aneurysm Not distinguished	No
Tatari	2022	Prospective	RVOT patch	AP	72 patients	Mean 13.48 ± 7.38 years	None: this was a histologic analysis study	–	–	–	Yes, 72 samples
Gluck	2020	Retrospective	Various congenital heart defects	GA-treated Cryopreserved Homograft Pericardium	276 patches in 134 patients	Mean 5.05 yrs (3 days, 12.42yrs) Median: 5.29 years	Significant valvular stenosis/regurgitation, significant residual shunt or bowing causing hemodynamic obstruction, significant vessel stenosis or aneurysm, or any adverse event leading to patch reop/explant/cath intervention	12 (9.0%) reoperations related to patch 18 (13.4%) reinterventions at patch sites	Patch failure-free survival: –5 years: 85.8% –10 years: 79.0%	9 (6.7%), none due to patch	Yes
Crawford	1986	Prospective	Various congenital heart defects (ASD, VSD, AV Canal, TOF, DORV, Pulmonary Atresia, RVOT)	Bovine pericardium	105 patients	Mean: 30.2 months (6–60)	–	No reported reinterventions	–Repeat roentgenograms showed no evidence of calcification	–12 (11.4%) dead intraoperatively, none related to patch –6 (5.7%) late deaths Unrelated to patch	Yes, 1 sample
Baird	2016	Retrospective	Various congenital heart defects	Photo-oxidized bovine pericardium (PhotoFix™)	490 patches in 364 patients	Mean 3.2 ± 1.6 yrs	–	5 (1%) reoperation prior to discharge unrelated to patch 5 (1%) catheterizations prior to discharge not distinguished 17 (4.4%) late reoperations, 1 related to patch 30 (8%) late catheterizations, 1 related to patch	–Freedom from all-cause reop at 5 years: 63.2 ± 4.3%	92% survival, no patch-related deaths	Yes, 8 samples
Kim	2016	Retrospective	Sutureless patch angioplasty for PA stenosis	Bovine pericardium	28 patients	Mean 60.9 ± 33.1 months	Pulmonary artery restenosis	1 (3.6%) reintervention 0 (0%) reoperation	–	Overall survival rates were 96.3%, 92.4%, and 92.4% at 1, 5, and 10 years, respectively. No patch-related deaths	No
Gustafson	1988	Prospective	TOF repair	Dacron	36 patients	2–37 months	–	0 (0%)	Note that outcome reporting focused comparing two surgical techniques, rather than evaluating graft material performance	–	No
Simon	2017	Retrospective	TOF repair	Dacron	94 patients	7.9 ± 3.4 yrs	RVOT stenosis	6 (6.4%) catheter-based total 5 (5.3%) reoperations Not distinguished	Note that outcome reporting focused comparing two surgical techniques, rather than evaluating graft material performance	2 late deaths not distinguished 97.8% 10-year survival	No
Fraint	2016	Retrospective	PA augmentation	CorMatrix, AP, bovine pericardium, cryopreserved pericardium, homograft material, ePTFE	221 patients, comparison of an ECM cohort (*n* = 48) to a standard patch material cohort (*n* = 173)	Median –472 days for ECM –1190 days for standard patch materials	Various	Total Reintervention + Reoperation: unspecified –ECM: 14 (29%) – 5 surgical, 9 catheterization –SP: 67 (39%) – 35 surgical, 32 catheterization	–	ECM: 3 (6%) SP: 11 (6%)	No
Quarti	2011	Prospective	Various cardiac tissue repairs (Cardiac tissue repair, PA arterioplasty, valve leaflet extension)	CorMatrix	27 patches in 26 patients	Mean 13.2 months	Stenosis	1 (3.7%) reoperation Due to peripheral stenosis		0%	No
Scholl	2010	Retrospective	Various congenital heart defects	CorMatrix	40 patients	Mean: 7.85 (0.5,24) months	–	1 (2.8%) reoperation 1 (2.8) catheter reintervention Not distinguished	–	5 deaths, 4 operative (10%), 1 late (2.8%)—all unrelated to the patch	Yes
Naik	2017	Retrospective	Non-transannular repair of TOF	CorMatrix, Bovine Pericardium	21 patients	CorMatrix Group: 28 ± 12.6 months Bovine Pericardium Group: 50.05 ± 17.6 months	–	–	Reports on 3D RVEF, Average RV Global Longitudinal Strain, and Tricuspid Annular Plane Systolic Excursion	–	No
Witt	2013	Retrospective	Various congenital heart defects	CorMatrix	37 patients	411 (62, 757) days	RVOT obstruction Stenosis	2 reoperations related to patch 1 reoperation unrelated to patch	–	4 (10.8%), none related to patch	Yes
Ashfaq	2017	Retrospective	Various congenital heart defects	CorMatrix	202 patients	Mean: 1492 days	Leaflet failure Residual shunting	10 reoperations related to patch integrity	–	7 (3.47%), unrelated to patch	Yes
Zaidi	2014	Retrospective	Valvoplasty	CorMatrix, Autologous Pericardium	57 patients	Not follow-up, but *in situ* time: Median time in situ for Mitral valvuloplasty group: CorMatrix: 64 (5–261) days AP: 1010 (14–1506) days Median time in situ for Aortic valvuloplasty group: 63 (49–198) days CorMatrix: 1747 (6–6047) days	Various need for explantation	18 explants 7/18 due to valve failure 11/18 due to tissue substitute	–	1 death not distinguished	Yes, 18 samples
Haney	2021	Retrospective	Various cardiac tissue repairs	CorMatrix	408 patches in 309 patients—180 PA patches	Median 3.9 yrs (3 days 7.4 yrs)	Various	Of PA Patches: 4 (2%) surgical reinterventions 22 (12%) percutaneous reinterventions	–	39 (12.6%), no deaths related to patch	No
Woo	2016	Retrospective	Valvoplasty, Aterioplasty	CorMatrix	12 patches in 11 patients	Mean time in situ*:* 518.6 (77, 1294) days	–	–	–	–	Yes, 12 samples
Rosario-Quinones	2015	Retrospective	Various cardiac tissue repairs (PA, Mitral valve, Aorta)	CorMatrix	25 patients	Time in situ*:* 9wk–13mo	–	6 (24%) reoperations	–	2 (8%) deaths, not distinguished	Yes
Hopkins (Pt. 1)	2014	Prospective cohort	Various cardiac tissue repairs	MatrACELL	120 patches in 108 patients	Median 687 (1, 842) days	Various	7 (5.83%) reoperations and 11 (9.17%) catheterizations, but none attributable to patch material	–	1.8%, none related to patch	No
Hopkins (Pt. 2)	2014	Retrospective cohort	Various cardiac tissue repairs	Cryopreserved PA autografts, synthetics	101 patches in 100 patients	–	stenosis, aneurysm, pseudoaneurysm	14 (14%) reoperations due to patch failure	–	None related to patch	No
Lofland	2012	Retrospective	Various cardiac tissue repairs (PA, RVOT, RPA, LPA, MPA, Aorta, SVC)	MatrACELL	46 patches in 44 patients	Not given, reported maximum follow-up of 22 months	–	1 elective patch removal	–	3 (6.82%), unrelated to patch	Yes
Murin	2021	Prospective	Branch PA augmentation	MatrixPatch	81 patients	Median 20mo, IQR (10.2–30.2)	–	probability of freedom from reop/reint: 1 year: 85.8% 2 years: 78.7% Not distinguished	–	No patch-related deaths	Yes
Bell	2019	Prospective	Various cardiac tissue repairs (VSD, ASD, AVSD, Aortic Arch, DORV, TAPVD, SVC, IVC, PA)	CardioCel	195 patches in 135 patients	Median 39 (27–54) months	Various	12 (6.2%) interventions—6 catheter, 6 surgical Not distinguished 1- and 3-year freedom from reintervention rates of 94% and 93%, respectively	5 of 34 (3.6%) pulmonary artery patch implants required reintervention in < 365 days. No reinterventions were conducted in this group at > 365 days	6 (4.6%), none related to patch	No
Bell	2019	Retrospective	Various	CardioCel	501 patches in 377 patients	Median 31 (1, 60) months	Stenosis, obstruction, RVOT related	14 (2.8%) patches required at least one reintervention—9 catheter, 9 operative Not distinguished		11 (2.9%) deaths, 1 (0.3%) patch related	Yes
Neethling	2013	Prospective	Various cardiac tissue repairs (VSD, ASD, RVOT, TOF)	CardioCel	30 patients	Range of 6–48 months	–	0%	MRI assessments in 10 patients at 12 months revealed no detectable micro-calcification levels	5 (16.7%), none related to patch	No
Neethling	2020	Retrospective	Various cardiac tissue repairs	CardioCel	24 patients with follow-up	Median 7.2 (1.3–10.6) yrs	–	1 (4.17%), not reported as graft related	No surface thickening, structural leaks, calcification, thromboembolic events	2 late deaths, none related to patch	No
Prabhu	2017	Prospective	Various cardiac tissue repairs	CardioCel	140 patients	Mean in situ time: 249.6 (10, 428) days	Various	15 reoperations—not distinguished 6 explants, 2 related to patch		Not reported	Yes
Pavy	2018	Retrospective	Various cardiac tissue repairs (ASD, VSD, AVSD, RVOT)	CardioCel	101 patients	Mean 212 (4, 726) days	Various	5 (4.9%) graft-related reoperations 2 unrelated reoperations	4 of 5 patches requiring reintervention were utilized for aortic angioplasty. 1 of 5 patches requiring reintervention was utilized for right coronary cusp replacement	4 (3.9%) none related to patch	Yes, 5 samples
Hongu	2022	Retrospective	PA reconstruction, Aortic Valve Plasty	TEVG	7 patients	median: 14.4 (3–39.6) months	Aneurysm, Degeneration, Infection, Stenosis	0 (0%) graft-related reinterventions	One patient demonstrated bronchus compression-induced PA restenosis	0 (0%)	
Nakatsuji	2021	Prospective	PA reconstruction	TEVG	4 patients	18.3 (4, 48) months	–	0 (0%) graft-related reinterventions		0 (0%)	Yes
Kato	2016	Prospective	PA reconstruction	TEVG	1 patient	9 months	–	0 (0%) graft-related reinterventions	This report describes the first successful clinical application of the Biotube for pediatric PA patch augmentation	0 (0%)	No
Fujita	2020	Prospective	PA reconstruction	TEVG	2 patients	–	–	–	Various mechanical characterizations	–	Yes

*TOF* tetralogy of fallot, *PA* pulmonary artery, *AP* autologous pericardium, *PTFE* polytetrafluoroethylene, *GA* glutaraldehyde

‘-’indicates not reported

In summary, autologous pericardium has the largest body of literature with long-term rates of reintervention of 5–15%. Glutaraldehyde fixation improves handling characteristics of the patch and may reduce the risk of aneurysm formation but increases the risk of subsequent calcification. Intriguing results demonstrate the possibility for autologous pericardium to serve as a matrix for tissue formation which resembles native tissue. Decellularized cardiovascular allograft patches (Matracell) and tissue engineering approaches have promising short-term results which support the concept of in situ tissue engineering approaches with extracellular matrix scaffolds to promote a favorable wound healing response and support cell infiltration, migration, and vascularization. However, further longitudinal study and histologic analysis are warranted. Improved tissue processing techniques of xenograft pericardium (Cardiocel) has resulted in decreased rates of calcification and need for intervention compared to prior use of bovine pericardium.

A general overview of the reported benefits and disadvantages of each patch type is shown in Table 2. A more detailed overview of each patch type and analysis of the reported clinical studies is provided in the subsequent sections.

**Table 2:** Reported advantages, disadvantages, and costs of different patch materials when applied to RVOT reconstruction and pulmonary artery arterioplasty

Material	Advantages	Disadvantages	Cost*
Autograft			
Autologous pericardium	–Non-antigenic –Potential for growth and remodeling –Limited calcification if not fixed with glutaraldehyde	–Limited Availability –Limited mechanical strength with possible risk of aneurysm formation if not fixed –Difficult to handle if not fixed –Calcification if fixed with glutaraldehyde	No cost
Allograft			
Cryopreserved pericardium or pulmonary artery	–Improved availability over autografts	–Antigenicity –Calcification if fixed with glutaraldehyde –Difficult to handle if not fixed	~ $5,000
Decellularized allogeneic pulmonary artery patches (MatrACELL)	–Reduced antigenicity compared to homograft –Reduced calcification compared to homograft	–Long-term study limited	~ $5,000
Xenograft			
Bovine Pericardium	–Availability –Easy to handle –Demonstrated potential for re-endothelialization (CardioCel™)	–Antigenicity –Necessity of pre-implant tissue fixation which increased risk of calcification	Cardiocel $1,400 (4 × 4 cm) $1,700 (5 × 8 cm) $2400 (RVOT)
Porcine small intestine submucosal extracellular matrix (SIS-ECM)	–Availability –Easy to handle	–Antigenicity despite rigorous processing –Not shown to act as a scaffold for native revascularization	$1,395 (2 ply 4 × 7 cm)
Synthetic			
Expanded polytetrafluoroethylene	–Availability –Easy to handle –Mechanical stability	–Non-biodegradable –Chronic foreign body response to implant –No potential for native revascularization	$500–800
Tissue Engineered	–Non-antigenic –Preliminary findings of tissue remodeling –Possibility for growth and remodeling	–Limited availability	N/A

*Costs as listed per Nationwide Children’s Hospital Institutional Cost Center

### Autologous Patches

**Fig. 2:**
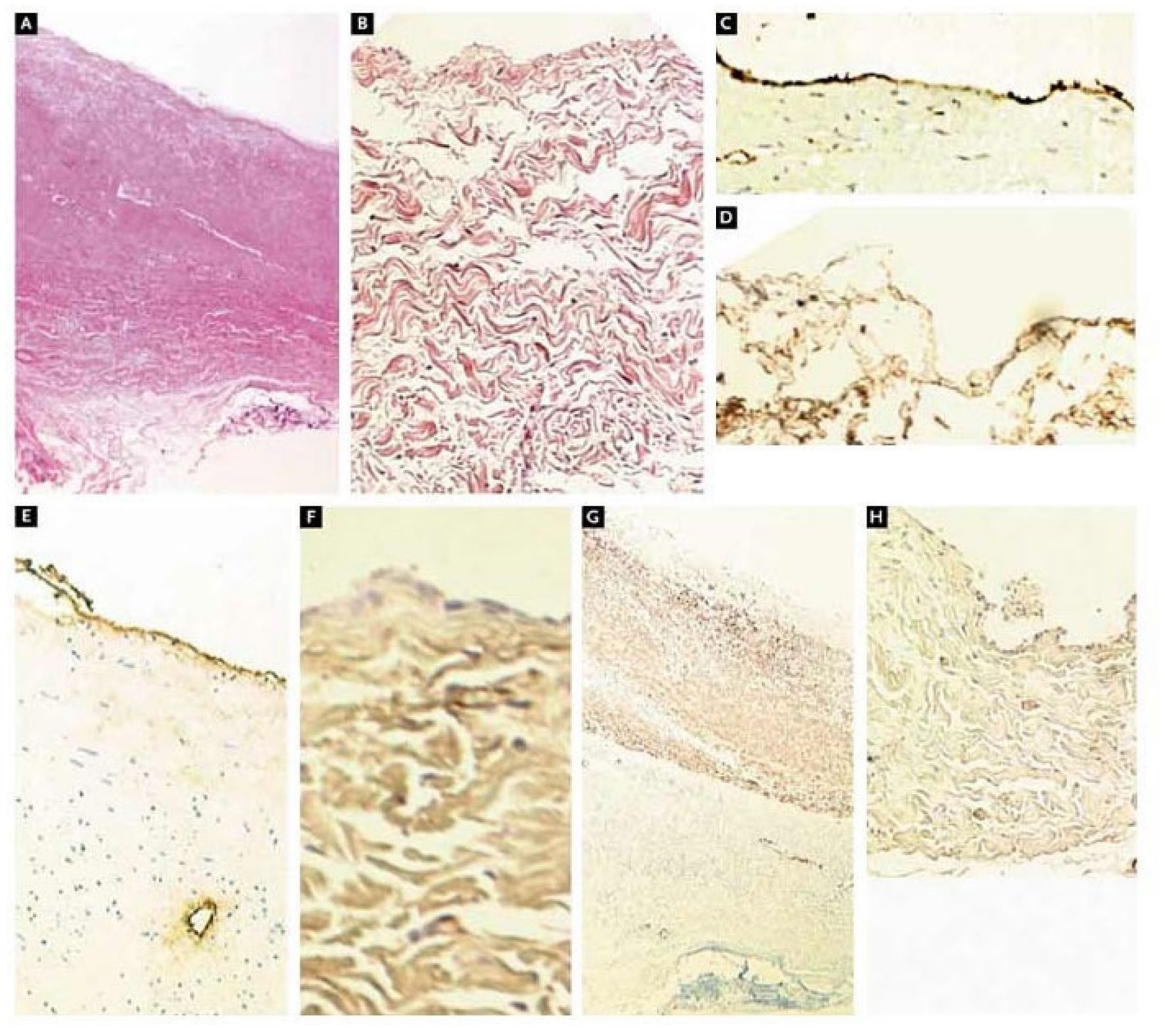
The Histology of a Pulmonary Artery Reconstructed with the Use of Autologous Pericardium, as Compared with the Histology of Native Pericardium. Hematoxylin and eosin staining is shown in the pulmonary artery (Panel **A**) and native pericardium (Panel **B**). Staining for CD34 is shown in the pulmonary artery (Panel **C**) and native pericardium (Panel **D**). Staining for factor VIII is shown in the pulmonary artery (Panel **E**) and native pericardium (Panel **F**). Staining for smooth muscle α-actin is shown in the pulmonary artery (Panel **G**) and native pericardium (Panel **H**) [3]

Autologous pericardium was the first non-synthetic material to be compared to synthetic materials for use in the reconstruction and repair of the RVOT and pulmonary arteries [17]. In the early 1970s, multiple studies confirmed the superiority of autologous pericardium over a previous clinical standard of formalinized polyvinyl (Ivalon) and indicated similar performance to polytetrafluoroethylene (PTFE) [18, 19]. Autologous pericardium is a logical choice, as it is free of cost, non-immunogenic, and can be sourced directly from the patient during surgery [20–22]. However, a primary limitation of autologous pericardium is lack of availability. Many CHDs require staged surgical procedures and a lack of available tissue for surgery is not an uncommon occurrence [17]. In its natural, unfixed state, autologous pericardium also tends to shrink or curl, which renders it difficult to handle surgically. Additionally, unfixed autologous pericardium may progressively dilate in situ, predisposing patches to aneurysm [20]. An example of RVOT patch aneurysm formation is shown in Fig. 1. Importantly, the significance of the potential complication of dilatation is not universally agreed upon, and some researchers maintain that the benefits of fresh autologous pericardium outweigh the potential risks [23]. A recently published study by Tatari et al. demonstrated outcomes of fresh autologous pericardium RVOT patches at a mean follow-up interval of 13.48 ± 7.38 years [23]. Of a 72-patient cohort, 53 (73.6%) showed no RVOT dilatation, 17 (23.6%) showed mild RVOT dilatation, and 2 (2.8%) had RVOT aneurysms [23]. In the same study, histologic analysis confirmed the positivity of explanted patches for CD31, CD34, smooth muscle alpha-actin, and von Willebrand factor (VWF) [23]. In a separate study, Hibino et al. showed histologically at three-year post-implant that autologous pericardium used for pulmonary artery arterioplasty differentiates to resemble tissue of the pulmonary artery (Fig. 2) [3]. This suggests that unfixed autologous pericardium has improved potential to differentiate into vascular wall tissue, at the risk of RVOT aneurysm.

**Fig. 3:**
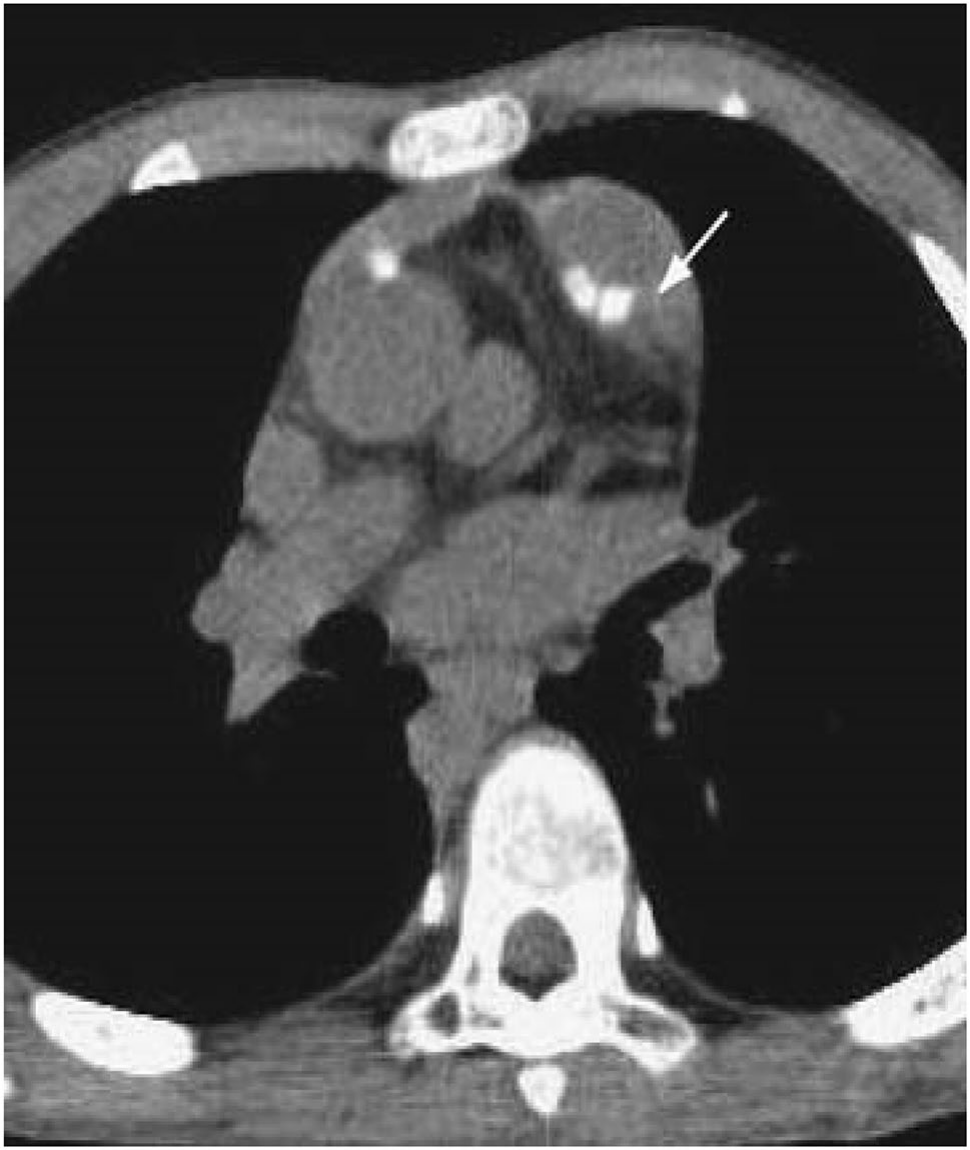
Non-contrast computerized tomography scan demonstrating calcification of an RVOT patch [1]

Glutaraldehyde fixation is a method of treating autologous pericardium that makes it easier to handle and improves material stiffness, reducing the risk of aneurysm [20]. This tissue processing technique can be performed intraoperatively, resulting in little delay and no need for separate procedures to harvest and implant the material [20]. In a comparison between saline- and glutaraldehyde-treated pericardial patches used in RVOT reconstruction, Messina et al. found reduced RVOT dilatation in the glutaraldehyde group 6 months after the operation [24]. The severity of dilatation (+ 0 to + 4) was assessed relative to the size of the aortic valve annulus [24]. Lee et al. found that longer fixation times in glutaraldehyde resulted in improved extensibility and better fixation compared to saline-treated controls, but a higher risk of long-term calcification [25]. An example of RVOT patch calcification is shown in Fig. 3. Tatari et al. evaluated 72 untreated autologous pericardial RVOT patches histologically, finding 7 (9.72%) were mildly calcified and 7 (9.72%) were moderately calcified at a mean follow-up of 13.48 years [23]. No severe calcification was noted. In terms of overall clinical performance, one study with a median follow-up time of 3.7 years compared a cohort of unprocessed autologous pericardial RVOT patches and glutaraldehyde-fixed autologous pericardial patches to a cohort of alternative patch materials covered later in this review, including homograft pericardium, bovine pericardium, and porcine small intestinal submucosa extracellular matrix (ECM) [26]. Autologous pericardial RVOT patches had a 7.3% reintervention rate, as opposed to a 15.9% rate in the total cohort [26].

### Homograft (Allograft) Patches

Homograft pericardium, sourced from genetically unrelated human donors, is considered inferior to autologous pericardium by performance metrics, but its increased availability makes this material a favorable option [27]. Homografts exhibit handling properties very similar to those of autologous pericardium and are often treated with glutaraldehyde to improve tissue handling characteristics, decrease the likelihood of in situ aneurysm, and reduce antigenicity [27, 28]. Unfortunately, calcification is a common complication associated with homograft pericardium and is usually attributed to inflammatory processes incited by transplant antigens [11]. Gluck et al. reported their institutional experience with cryopreserved homograft pericardium in a study with a mean follow-up time of 5.05 years and range of 3 days to 12.41 years [27]. Twelve patients (8.96%) underwent reoperation and 18 patients (13.4%) underwent catheter interventions at sites of patch implantation [27]. In another study, when compared to multiple other standard patch materials including decellularized porcine small intestine submucosa (CorMatrix™), bovine pericardium, and autologous pericardium, homograft patches were found to have the highest rate of surgical or catheter-based reintervention, 18.5%. This outpaced the study average of 15.9% and the autologous pericardium group rate of 7.3%, although these differences were not statistically significant [26]. Hopkins et al. demonstrated a 20.3% failure rate for cryopreserved homograft patches compared to 0% and 4.9% for decellularized allogeneic pulmonary artery patches (MatrACELL™) and polytetrafluoroethylene (PTFE) patches, respectively, over an approximate three-year follow-up period [11].

#### Decellularized Cardiovascular Allograft Patches

MatrACELL is the brand name for a decellularization process approved for use in the preparation of cardiovascular allografts (LifeNet Health™). MatrACELL patches utilized for RVOT and pulmonary artery reconstruction specifically are composed of human pulmonary artery extracellular matrix (ECM) and are supplied in three forms: thin patches, thick patches, and hemipulmonary arteries [11, 29]. The decellularization process uses an anionic, non-denaturing detergent (sodium lauroyl sarcosinate) to solubilize and remove the donor cells and a recombinant endonuclease to degrade donor DNA/RNA followed by removal of detergent and cellular remnants [11].

Lofland et al. studied a cohort of 46 patches and found no patch-related deaths or patch-related complications [29]. The same group undertook a prospective clinical study of MatrACELL patch performance and compared these results to a retrospective study of cryopreserved pulmonary artery homograft patches and synthetic patches [11]. At a median implant duration of 687 (Range: 1–842) patient days, 0% of patches in the prospective cohort required reintervention. This is significantly different from the performance of patches in the retrospective cohort, in which 14% required reintervention (*p* < 0.0001) [11]. Reinterventions in the retrospective group occurred at a mean duration of 194 ± 104 days, with the latest occurring at postoperative day 477, a shorter timeline than the median implant duration of the prospective group [11]. The two cohorts were anatomically and surgically similar, but a comparison of causes for intervention was not reported, representing another potential limitation [11]. When conducted, imaging studies (roentgenograms, echocardiograms, computerized tomography) showed no evidence of patch calcification in the MatrACELL cohort, and calcification was never observed at subsequent surgical staged procedures [11]. There was no universally applied modality to detect calcification, which is a limitation for these findings.

### Xenograft Patches

#### Bovine Pericardium

Bovine pericardium, like homograft pericardium, is a patch material introduced with the primary advantage of increased availability. Outside of logistical considerations, bovine pericardium exhibits low antigenicity and has favorable surgical characteristics, including reduced elasticity, appropriate stiffness, and smooth, even edges [21, 30]. Unfixed bovine pericardium becomes inflamed and undergoes partial digestion. As a result, multiple cross-linking methods have been tested to reduce antigenicity and enhance mechanical strength [14, 30]. Two methods in particular, glutaraldehyde fixation and photo-oxidation have been used to prepare bovine patches tested in clinical trials [14, 31, 32]. Fixation, especially via glutaraldehyde, has been shown to lead to increased calcification, a well-known consequence of the use of bovine pericardium [14]. Calcification likely develops via recipient inflammatory responses incited by transplant antigens, since donor cell remnants remain in the tissue even after processing [11, 33]. A third, newer, method of processing bovine pericardium prior to patch implantation is the ADAPT TEP™ method (AdmedusRegen Pty Ltd™) [33]. This method still employs glutaraldehyde for tissue fixation but contains additional steps to limit downstream calcification [33].

##### Glutaraldehyde Fixation of Bovine Pericardium

When compared to a range of glutaraldehyde concentrations from 0.2 to 1%, fixation in 0.5–0.6% glutaraldehyde results in the most favorable material characteristics for bovine tissue, including material properties and post-implantation resistance to calcification relative to alternative glutaraldehyde concentrations [25]. When compared to bovine tissue not treated with glutaraldehyde, a greater foreign body response and increased calcification are seen in glutaraldehyde-treated tissue. During clinical application in various CHD surgical procedures, including RVOT and pulmonary artery reconstruction, Crawford et al. found this material to be flexible and easy to suture and saw no patch calcification via repeat roentgenograms in all patients over a mean follow-up of 30.2 months [34]. A small retrospective study (*N* = 28) with a mean follow-up of 60.9 months saw favorable performance from bovine pericardial patches with a 3.6% reintervention rate and a 0% reoperation rate [35]. In a study comparing bovine pericardium to ECM patches for non-transannular repair of tetralogy of Fallot, no significant difference was found between the two patch types when evaluating three-dimensional right ventricular ejection fraction and tricuspid annular plane systolic excursion values [21].

##### Photo-fixation of Bovine Pericardium

Photo-fixation is an alternative to glutaraldehyde fixation for bovine pericardium preparation [31]. This tissue processing method utilizes dye-mediated photo-oxidation to cross-link pericardial collagen, as opposed to chemical cross-linking, which occurs during glutaraldehyde fixation. Tissue is treated with a photo-active dye and then exposed to specific wavelengths of light [31]. PhotoFix™ pericardium is reported to be non-immunogenic, non-calcifying, non-cytotoxic, and enabling of endothelialization [31]. Glutaraldehyde treatment of tissue, on the other hand, improves biostability but leaves behind residual aldehydes, which increase the risk of subsequent calcification [31]. Baird et al. reported a retrospective study of PhotoFix™ bovine pericardial patches (*N* = 490). There was a 92% survival rate at a mean follow-up of 3.2 ± 1.6 years, and no deaths were related to failure of the patch material. The Kaplan–Meier estimate of survival at 5 years was 90.6 ± 1.7%, and the rate of freedom from all-cause reoperation at 3 and 5 years was 85.6 ± 2.1% and 63.2 ± 4.3%, respectively. They found that 47 patients (12.9%) underwent late reoperation or catheter reintervention. One reoperation (0.2%) was conducted due to fracture of a heavily calcified patch and one catheter reintervention (0.2%) was due to a developing patch aneurysm [31]. Patch material was explanted from 8 patients with a mean in situ time of 20 months and showed mild to moderate inflammation with variable calcification. Neointima formation was mild or less in all cases [31].

##### ADAPT TEP™ Tissue Processing of Bovine Pericardium

CardioCel*™* is bovine pericardium that undergoes a proprietary anticalcification tissue engineering process (ADAPT TEP™) (LeMaitre Vascular©) [33]. While still employing a low-concentration glutaraldehyde solution to fix and strengthen the material, ADAPT TEP™ attempts to avoid the downstream complications of glutaraldehyde fixation by utilizing non-glutaraldehyde sterilization and storage methods [33]. There are multiple steps undertaken to reduce cytotoxicity, including the removal of lipids, cells, cell remnants, nucleic acids, and galactosyl epitopes and the eventual storage in a glutaraldehyde-free solution [28, 33]. CardioCel™ has been reported to have favorable intraoperative characteristics, including uniform thickness, flexibility, elasticity, ease of surgical seating, and good suture retention [33, 36]. Prabhu et al. noted durability and strength like that of autologous pericardium [14].

Multiple clinical studies have shown strong performance of CardioCel™ patches when applied to RVOT or pulmonary artery reconstruction [14, 28, 33, 37]. A study by Bell et al. that evaluated 195 patch implants in a variety of anatomic locations at a median follow-up of 39 months reported 1-year and 3-year freedom from reintervention rates of 94% and 93%, respectively [33]. The reintervention rate over the full duration of the study was 6.2% [33]. However, pulmonary artery patches required reintervention more frequently than patches in any other anatomic location, with a rate of 3.6% over the course of the study [33]. The same group evaluated a larger cohort of patients (*N* = 501) and found similar results, with 96% of patients free from reintervention at 5 years [28]. Pavy et al. conducted a study of CardioCel™ patches in a variety of anatomic positions with a median follow-up of 212 days [37]. This group demonstrated a 0% patch-related mortality rate and 4.9% patch-related reintervention rate [37]. None of the patches requiring reintervention were utilized for RVOT or PA reconstruction [37].

A study conducted by Prabhu et al. histologically evaluated six CardioCel™ patches with in situ lifetimes of 10, 67, 134, 272, 292, and 428 days. No explants displayed patch breakdown, and the architecture of laminated native collagen fibers was maintained in all [14]. Neointima development had begun in all but the 10-day explant, and endothelialization was observed in the peripheral portion of the 292-day explant, which was separately explanted at 502 days [14]. Additionally, in the 502-day peripheral portion explant, fibroblasts had produced bundles of collagen fibers, an important indicator of early tissue remodeling. The five earlier explants evaluated in this study showed fibroblast invasion that appeared to increase with the age of the explant, but no new collagen deposition [14]. Inflammatory infiltrate was found to extend into granulation tissue forming on the parietal layer of each graft but did not penetrate into any of the grafts [14]. Lastly, calcification occurred in one of the six explants in the space between the layer of granulation tissue and the parietal surface of the graft. The authors suggested this was indicative of postoperative pericarditis rather than a foreign body reaction to the graft [14]. No explants demonstrated any indication of remodeling into a 3-layered vessel wall, although the short timeline of the study could have limited this process. CardioCel™ patches implanted into different anatomic regions have different histologic outcomes, so further studies will need to be conducted with the RVOT or pulmonary arteries specifically in mind to evaluate effectiveness in these locations [37].

#### Decellularized Equine Pericardium

*Matrix Patch™* (Auto Tissue GmbH Berlin™) is a decellularized equine pericardium with a single clinical study reporting performance. In this study, 119 patches were used to augment the branch pulmonary arteries of 81 patients who were followed for a median of 20 months [38]. There was no patch-related death, and the probability of freedom from reoperation or stent implantation at 12 and 24 months was 85.8% and 78.7%, respectively [38]. Three Matrix Patch™ tissue samples were explanted for histopathological analysis at 6-, 20-, and 22-month post-implantation. *Von Kossa* staining was negative in each of the three patches, confirming the absence of calcification [38].

#### Decellularized Porcine Small Intestine Submucosal Extracellular Matrix (SIS-ECM CorMatrix™)

For the past 15 years, CorMatrix™ has been applied to RVOT and pulmonary artery reconstruction. CorMatrix™ demonstrates favorable material qualities, including strength, ease of surgical handling, and a resistance to calcification and shrinkage [10, 12]. Unlike most other patch materials, SIS-ECM does not stretch; thus, mismatches between patch and blood vessel dimensions must be overcome by patch oversizing rather than stretching [10]. Much short-term data has proven CorMatrix™ to be an effective patch material able to compete with prior clinical standards.

Haney et al. evaluated the performance of 408 CorMatrix™ patches in 309 patients, with 259 patches specifically implanted into the RVOT or pulmonary arteries. With a median follow-up of 3.9 years, there were 39 deaths (12.6%) with 0 related to patch failure [10]. 5-year freedom from reoperation was 96%, and 5-year freedom from any form of reintervention was 88% [10]. Of the 180 pulmonary artery patches, 4 (2%) required surgical reintervention and 22 (12%) required percutaneous reintervention at 5 years [10]. The main failure modalities were pseudoaneurysm, aneurysm, delamination, and stenosis. The incidence of calcification was not reported as there were no studies conducted to evaluate this complication specifically [10]. Numerous small studies with shorter follow-up periods have shown similar, if not more favorable, results [12, 13, 21, 39, 40]. When compared directly to standard patch materials, including autologous pericardium, homograft and xenograft pericardium, and synthetic materials, no significant difference has been reported in outcomes for patients receiving CorMatrix™ patches [21, 41]. Fraint et al. compared CorMatrix™ patch patients (*N* = 48) to a standard patch material cohort (*N* = 173) composed of autologous pericardium, native PA tissue, non-native pericardial tissue, pulmonary and aortic homografts, and ePTFE [41]. This study demonstrated identical mortality rates of 6%, and total reintervention rates of 29% for CorMatrix™ as opposed to 39% for standard materials. Of note in this study, standard patch material patient median follow-up to track reintervention rates was 1190 days, whereas ECM patient median follow-up was 472 days, leaving additional time for complications to arise in the standard patch material group, possibly skewing reported data [41].

**Fig. 4:**
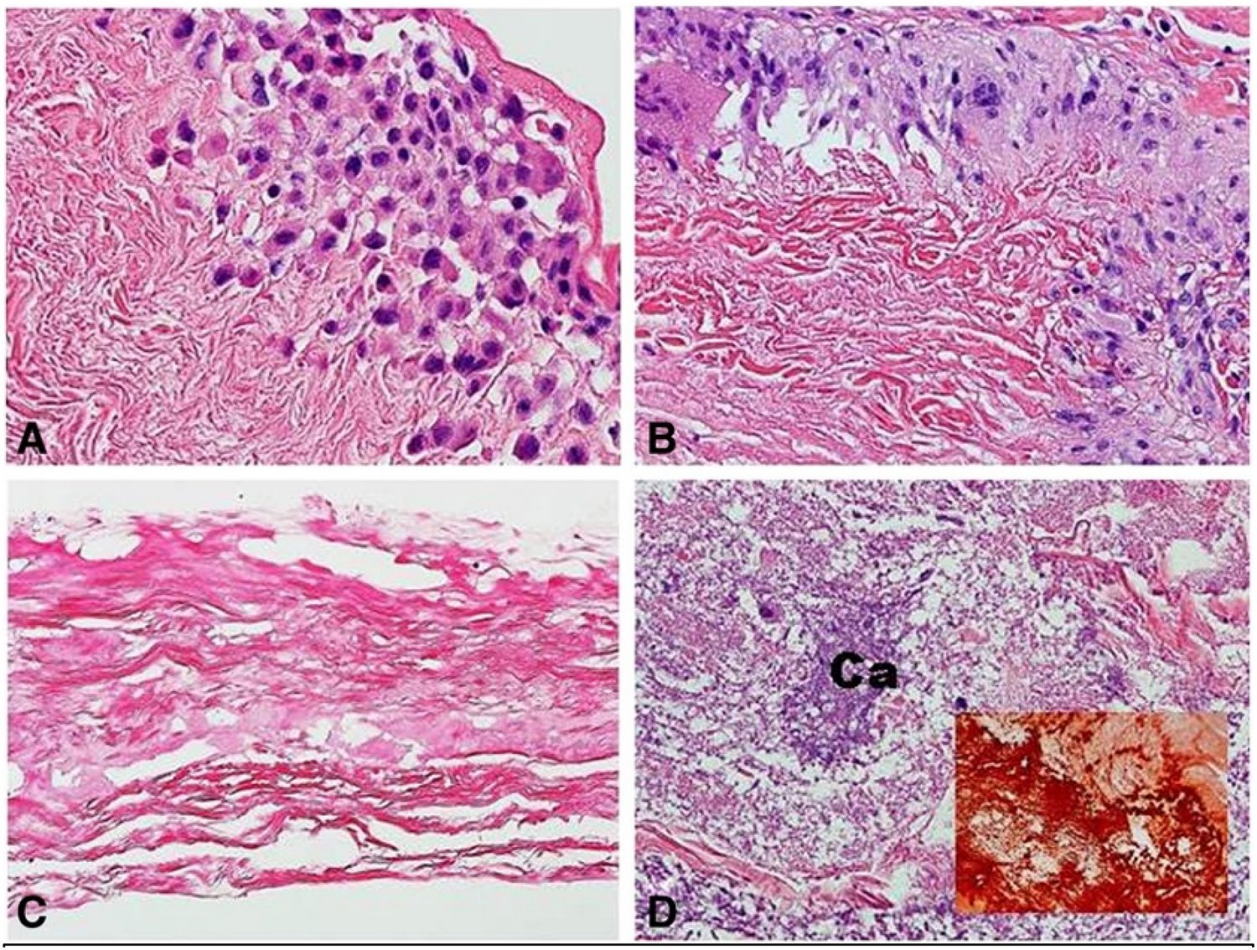
**A** CorMatrix™ collagen infiltrated by lymphocytes and plasma cells; **B** relatively intact CorMatrix™ collagen with prominent surrounding foreign body giant cell reaction; **C** degenerative changes of CorMatrix™ collagen; and **D** more severe degeneration of fibers with calcification (Ca); inset: calcifications are confirmed by the alizarin red stain (H&E stain, × 200) [2]

Few studies have been performed in patients to evaluate histologic changes of patches post-implantation to determine the degree of tissue integration and recellularization of this patch material [2, 42]. Woo et al. evaluated 12 explanted CorMatrix™ specimens from 11 patients that had been present in situ for an average of 518.6 days and found no histologic evidence that these patches acted as a scaffold for reconstitution of native cardiovascular structures (Fig. 4) [2]. Six of the twelve specimens in this study were explanted for reasons other than patch failure, so these results represent outcomes for adequately performing patches in addition to failed patches [2]. Similar results were reported by Zaidi et al. after a median in situ lifetime of 3 and 6 years for CorMatrix™ reconstruction of mitral and aortic valves, respectively [42]. The relevance of this study to CorMatrix™ behavior in RVOT reconstruction is unknown [42]. The studies conducted by Woo et al. and Zaidi et al. are limited by small sample sizes, short patch lifetimes, differing criteria for declaring patch remodeling, and differing surgical applications.

### Synthetic Patches

#### Polytetrafluoroethylene and Expanded Polytetrafluoroethylene

Polytetrafluoroethylene (PTFE, Teflon) and expanded polytetrafluoroethylene (ePTFE, Gore-Tex) are materials that have been used historically for RVOT and pulmonary artery patches. These microporous materials have relatively favorable biocompatibility and are associated with less fibrosis than prior synthetic materials, such as Ivalon [22]. PTFE was one of the first materials used for RVOT and pulmonary artery patch reconstruction, and it continues to be used today. In the short term, PTFE appears to perform as well as autologous pericardium [22]. In an early study, PTFE was found to perform similarly to autologous pericardium in terms of rate of aneurysm formation [18]. Hopkins et al. reported a study in which PTFE patches had lower failure rates than cryopreserved homograft patches (4.9% vs. 20.3%, respectively) [11]. No clear data were available detailing the performance of Gore-Tex patches applied to RVOT or pulmonary artery patch reconstruction.

#### Polyethylene Terephthalate

Polyethylene terephthalate (Dacron) is a synthetic material favored for its non-distensibility. Its low porosity with associated pseudointima formation has resulted in its use being largely abandoned in congenital heart disease surgical repair in favor of ePTFE. Its performance has not been extensively reported in RVOT reconstruction. One study (*N* = 36) showed no mortality or aneurysm formation in any patient over a follow-up of 2–37 months [43]. Simon et al., in a comparison of surgical techniques for tetralogy of Fallot palliation, demonstrated a freedom from reoperation rate at 10 years of 95.6% and 91.8% in limited transannular patch and annular sparing approaches, respectively [44]. Even though this study aimed to evaluate surgical techniques, late survival and freedom from reoperation rates of above 90% suggest that Dacron can be an effective RVOT patch material.

### Tissue-Engineered Patches

**Fig. 5:**
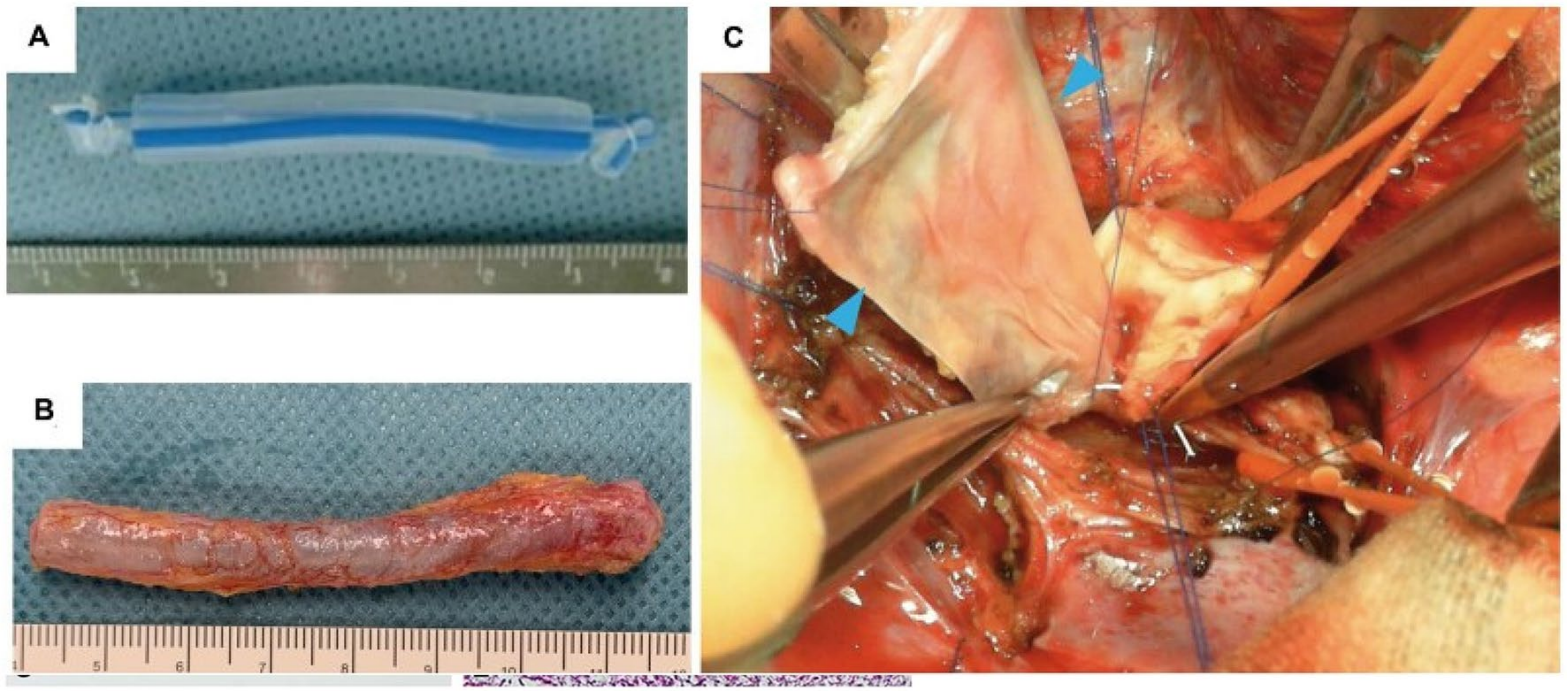
**A** A 19-Fr silicone drain tube with plugged-up lumen prepared as a mold of an in vivo tissue-engineered vascular graft. **B** Harvested molds are completely encapsulated with connective tissue. **C** In vivo tissue-engineered vascular graft patch (blue arrow) is implanted to treat pulmonary artery stenosis [5]

Autologous ECM constructs have been created, harvested, and applied for RVOT and pulmonary artery reconstructive surgeries using an in situ tissue engineering approach to develop tissue-engineered vascular grafts (TEVG) [5, 17, 45, 46]. Cylindrical 19-French silicone molds were embedded into the upper abdominal subcutaneous space and grown for 4 weeks to 4 months. TEVGs were harvested during the reconstructive surgery and cut open to be used as patch materials [5, 17, 46]. Histologic analysis prior to implantation characterized TEVGs as having smooth luminal surfaces and walls mainly composed of collagen fibers and some fibroblasts (Fig. 5) [5, 45].

This patch design was first clinically tested by Kato et al. who found no aneurysm formation or stenotic change of the patch after nine months in a single patient [17]. In another clinical study, researchers treated the TEVG patch with 70% ethanol to improve mechanical and surgical handling properties, similar to approaches tested with autologous, homograft, and xenograft pericardium [45]. Ethanol treatment dehydrates tissue and destroys cells ultimately “killing” the graft, but it is believed that tissue regeneration is enabled by the unique ECM structure that remains, even after such tissue preparation [45]. An analysis of the suture retention strength and burst pressure of this patch material indicated that it is safe to use for pulmonary arterioplasty [45]. Treatment of the TEVG material with 70% ethanol prior to implantation has continued in all subsequent studies thus far [5, 45, 46]. In a four-patient study, Nakatsuji et al. reported no mortality and satisfactory pulmonary dilation in all patients with an average follow-up period of 18.3 (range, 4–48) months [5]. One patient required right pulmonary artery augmentation due to bronchus compression-induced restenosis. In a separate manuscript reporting results from the same study, two additional patients underwent pulmonary artery augmentation via TEVG patches without complications noted [46]. Fibroblast invasion and collagen deposition, indicators of tissue remodeling, have been seen on histologic examination of TEVG patches without elastic fibers or smooth muscle cells in animal models, but no such clinical data exist, since no human patches have required explantation [5, 46]. The potential of TEVGs to enable neotissue development is a major driver behind this technology, but histologic analyses of patches post-explant from clinical studies are not available to evaluate this possibility further.

## Discussion

The current body of literature describing the performance of various patch materials for RVOT and pulmonary artery arterioplasty is growing. From the current literature, it is possible to derive an understanding of the advantages and limitations of individual patch materials. Autologous pericardium is an ideal patch material option, yet it is limited by restricted availability as well as its not insignificant risk of aneurysm. Tissue processing methods exist to bolster its mechanical properties (i.e., glutaraldehyde fixation), but such methods carry their own limitations, including increased immunogenicity and reduced growth capacity. Importantly, fresh autologous pericardium is favored for its ability to grow and differentiate to match its surroundings. Tatari et al. present convincing but inconclusive evidence to the superiority of fresh autologous pericardium over glutaraldehyde-fixed pericardium in the setting of RVOT patches [23]. The findings of growth potential, positive endothelial cell markers, vascular differentiation, and limited calcification all would suggest that fresh autologous pericardium has the potential to remodel and grow with the patient [23]. Homograft pericardium is anatomically like autologous pericardium but with the increased risk of immunoreactivity and tissue damage from cryopreservation and sterilization. Bovine pericardium, another popular option for patch material, has greatly improved availability over human-derived tissues, but its antigenicity provokes a chronic inflammatory response. Additionally, no studies have shown the degree of tissue remodeling to constitute neovessel formation.

Patch materials focused on supplying an ECM as a scaffold for vessel growth and remodeling hold promise. The ECM of native structures is a cell-produced scaffold that plays an essential role in tissue homeostasis [12]. Its role in signaling for proliferative, metabolic, and differentiative intracellular pathways lends utility to its use as a biomaterial [12]. The body exhibits a different response to ECM than to the synthetic, nondegradable materials, following a regenerative healing pathway that facilitates tissue remodeling rather than a proinflammatory pathway that predisposes the patch implant to fibrosis and scarring [12]. One proposed mechanism for these differing responses is a difference in macrophage subpopulations. Synthetic materials such as PTFE and Dacron encourage differentiation into the M1 phenotype, whereas the response to ECM shows a higher proportion of M2 macrophages [12]. M2 macrophages, in addition to resisting fibrosis and scarring, are capable of transdifferentiating into endothelial cells and accelerating the graft endothelialization process [12]. ECM is well conserved across various species and, thus, if well decellularized, should avoid the strong immune response seen with other implantable biomaterials [13]. Much of the promise of ECM patches for cardiac applications lies in their proposed ability to enable migration and proliferation of native cells, leading to the generation of viable neotissue [2, 21]. This process of constructive remodeling would present as the replacement of ECM patch material by native cells and organized, viable collagen [2].

Evidence with the use of decellularized porcine small intestine submucosal ECM (CorMatrix™) has shown variability in outcomes which may result from incomplete decellularization of constructs, differences in remodeling across patients, or variation in remodeling under differing hemodynamic environments. Evaluation by Woo et al. characterized the host response to CorMatrix™, finding significant inflammation, fibrosis, and necrosis of native tissue surrounding the implant [2]. These findings were consistent with a destructive host inflammatory response, contradictory to the proposed performance of ECM in situ [2]. These findings are similar to previous histologic findings in native tissue surrounding CorMatrix™ implants [42]. Two studies characterizing the histologic findings of explanted CorMatrix™ implants hypothesized that this significant inflammatory response arose in response to the xenogeneic nature of CorMatrix™ [2, 42]. Despite the rigorous process of decellularization and antigen removal, pig ECM expresses the Gal epitope, and humans express anti-Gal antibodies, which can serve as the impetus for an immune-mediated reaction and tissue degeneration [2]. Additional studies must be conducted to determine the ability of CorMatrix™ to facilitate tissue remodeling and neotissue growth.

Two very exciting new developments in the space of patch technology are MatraCell™ and potential tissue engineering solutions. These methodologies exhibit the potential to exceed the performance of competing patch types while navigating the issue of limited availability posed by autologous pericardium. Their success supports the development of ECM constructs which limit the immune response and perhaps promote a favorable regenerative reaction allowing for neotissue formation, improved long-term durability, and growth capacity. As an ECM patch, it is proposed to have many of the benefits expected of CorMatrix™ with the benefit of being allogeneic rather than xenogeneic. Western Blot testing of MatrACELL™ for human major histocompatibility complex showed no demonstrable residual transplant antigenic epitopes [11]. This represents a potential advantage of MatrACELL™ over CorMatrix™, which does not have such supporting evidence [2]. While MatrACELL™ is an exciting technology, further studies must be conducted to evaluate long-term efficacy.

There are several limitations in the reviewed body of literature worth noting. Most studies are focused on reporting their single-center experience with selection of patch material presumably based on surgeon preference and patch availability. The primary outcome of interest is typically reintervention-free survival. However, the usual retrospective study design makes controlling the indications for reintervention not possible, and the timing for reintervention may vary between institutions and clinicians. Patients may have significant stenosis but have not yet undergone reintervention for a variety of reasons. Patient size, disease severity, and surgical complexity are also shown to affect patch performance and differ across studies [27]. These differences make statistical comparisons between studies difficult.

### Conclusion/Future Directions

In future studies, several points are of interest that can improve the assessment of patch efficacy. A uniform evaluation and consistent criteria for intervention need to be applied across all patch types. While angiographic metrics are necessary for evaluating the degree of stenosis, assessment of angiographic measurements is challenging due to a lack of normative data regarding pulmonary artery size with no accepted z-scores. Additionally, there is no clear pressure gradient defined for intervention for RVOT stenosis or branch pulmonary arteries. In general, “significant stenosis is obvious when there are measurable gradients of > 20 to 30 mmHg across the stenotic area, when there is elevation of the right ventricular or proximal main pulmonary artery pressure to greater than one half to two-thirds of systemic pressure secondary to the more distal obstruction, or when there is relative flow discrepancy between the 2 lungs of 35%/65% or worse [47].” Differences in the mammalian regenerative capacity of the cardiovascular system have been shown to vary with age. Examples include the marked improvement in ventricular function that may occur after surgical correction of an anomalous left coronary artery arising from the pulmonary artery or in children with univentricular hearts who present enormous morphological changes after volume unloading [48]. The correlation of increased regenerative potential with age may be of critical importance when considering the possible regenerative capability of future patch materials. Differences in the mechanical cues imparted by the material and sensed by the resident cells may have a profound effect on patch remodeling and remodeling of the surrounding native vasculature. One such example is the variability of calcification of ePTFE patches depending on their location of implantation in patients with CHD [1]. Quantification of the differences in hemodynamics and mechanical stresses between patch materials applied to treat various congenital lesions will likely be of importance to better understand differences in outcomes. Although patches derived from human cells have a logical advantage in biocompatibility, it is difficult to test this possibility in preclinical studies. Uniform histologic analysis of explanted tissue for assessment of inflammation, fibrosis, necrosis, degenerative changes, eosinophil response, presence of foreign body giant cells, neovascularization, and calcification is key toward defining the immunologic response and capacity of patches to enable the regeneration of native vascular architecture [2].

## Supplementary Information

Below is the link to the electronic supplementary material.

Supplementary file1 (DOCX 137 KB)

Supplementary file2 (XLSX 17 KB)
