# Supplementary material for: Patch Materials for Pulmonary Artery Arterioplasty and Right Ventricular Outflow Tract Augmentation: A Review
Source: Pediatr Cardiol. 2023 May 7;44(5):973–95. doi: 10.1007/s00246-023-03152-7 (PMC10224813; doi:10.1007/s00246-023-03152-7)

**Supplemental Figure I (A)** Number of clinical studies of patch performance in PA arterioplasty or RVOT reconstruction by year. **(B)** Timeline of patch materials utilized in the clinic for RVOT reconstruction. Dates are according to the year of the first published clinical study of performance of a particular patch material.


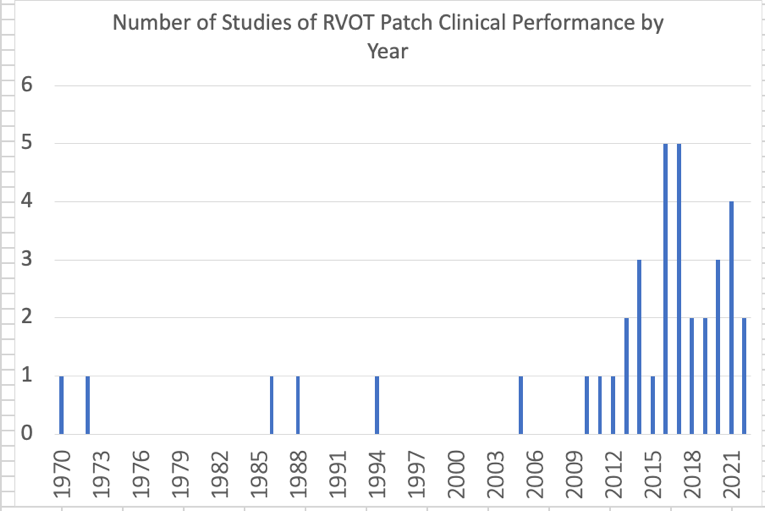

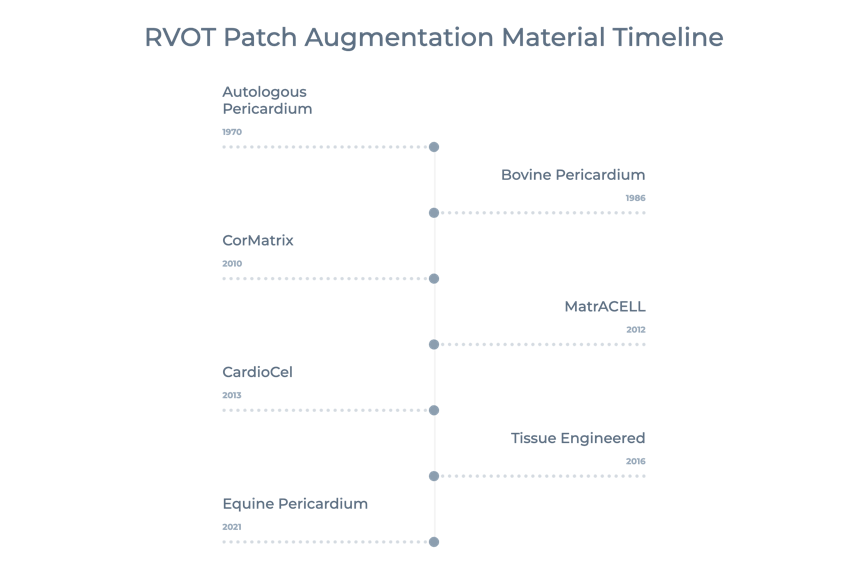

Supplement: Supplementary file 1 — Supplementary file1 (DOCX 137 KB) [file 246_2023_3152_MOESM1_ESM.docx]
